# Homotypic Entosis as a Potential Novel Diagnostic Marker in Breast Cancer

**DOI:** 10.3390/ijms24076819

**Published:** 2023-04-06

**Authors:** Ireneusz Dziuba, Agata M. Gawel, Paweł Tyrna, Jędrzej Machtyl, Monika Olszanecka, Andrzej Pawlik, Cezary Wójcik, Lukasz P. Bialy, Izabela Mlynarczuk-Bialy

**Affiliations:** 1 Department of Pathology, Faculty of Medicine, Academy of Silesia, 40-555 Katowice, Poland; mmid@wp.pl; 2 Histology and Embryology Students’ Science Association, Department of Histology and Embryology, Faculty of Medicine, Medical University of Warsaw, 02-004 Warsaw, Poland; agata.gawel@yahoo.com (A.M.G.); pawel.tyrna@gmail.com (P.T.); machtyljedrzej@gmail.com (J.M.); olszaneckamonika@gmail.com (M.O.); 3 Department of Physiology, Pomeranian Medical University in Szczecin, 70-111 Szczecin, Poland; pawand@poczta.onet.pl; 4 Amgen, Thousand Oaks, CA 91320, USA; 5 Department of Histology and Embryology, Faculty of Medicine, Medical University of Warsaw, 02-004 Warsaw, Poland

**Keywords:** homotypic entosis, cell-in-cell, breast cancer, Ki67, HER2, lymph node metastasis

## Abstract

Homotypic entotic figures, which are a form of “cell-in-cell” structures, are considered a potential novel independent prognostic marker in various cancers. Nevertheless, the knowledge concerning the biological role of this phenomenon is still unclear. Since breast cancer cells are remarkably entosis-competent, we aimed to investigate and compare the frequency of entoses in a primary breast tumor and in its lymph node metastasis. Moreover, as there are limited data on defined molecular markers of entosis, we investigated entosis in correlation with classical breast cancer biomarkers used in routine pathomorphological diagnostics (HER2, ER, PR, and Ki67). In the study, a cohort of entosis-positive breast cancer samples paired into primary lesions and lymph node metastases was used. The inclusion criteria were a diagnosis of NOS cancer, lymph node metastases, the presence of entotic figures in the primary lesion, and/or lymph node metastases. In a selected, double-negative, HER2-positive NOS breast cancer case, entoses were characterized by a correlation between an epithelial–mesenchymal transition and proliferation markers. We observed that in the investigated cohort entotic figures were positively correlated with Ki67 and HER2, but not with ER or PR markers. Moreover, for the first time, we identified Ki67-positive mitotic inner entotic cells in clinical carcinoma samples. Our study performed on primary and secondary breast cancer specimens indicated that entotic figures, when examined by routine HE histological staining, present potential diagnostic value, since they correlate with two classical prognostic factors of breast cancer.

## 1. Introduction

Cancer cells are characterized by aberrant metabolism, which is strengthened by starvation and hypoxic conditions that accompany the rapid growth of tumors [1]. Several cancer-related conditions, such as genetic instability or aberrant cancer cell proliferation, can be causative of certain special processes in tumors, such as homotypic entosis. Entosis is a process in which one cell invades another cell’s cytoplasm [2]. This phenomenon was first observed during in vitro studies using breast cancer cell lines, as well as mouse xenograft models [3], and was initially described as a new type of cell death, as the internalized cell can die by lysosomal lysis [2]. However, the internal cell can also escape degradation or may even divide inside the host cell (entotic mitosis) [2]. Homotypic entosis represents a subtype of “cell-in-cell” structures [4,5,6,7].

There are no automated methods for the identification of entosis. Entotic figures may be identified in tissue sections stained with hematoxylin and eosin (HE) using diagnostic histopathological criteria proposed by Mackay [8,9]. For counting entotic structures, at least four out of the six following features are required to be unambiguously identifiable: (i) the nucleus of the internalized cell; (ii) the cytoplasm of the internalized cell; (iii) a crescent-shaped nucleus of the engulfing cell; (iv) the cytoplasm of the engulfing cell; (v) the nucleus of the host cell; and (vi) an entotic vacuole between the engulfed and the outer cell. Entosis can also involve more than two cells. In the case of an entotic structure formed by three cells, the middle cell simultaneously acts as both an internalized and outer host cell [10].

Over the last years, there has been increasing interest in entosis as a potential new prognostic factor associated with worse prognosis for head and neck [11,12], tongue [13], bladder [14] and rectal cancer [12], as well as lung adenocarcinomas [8,9] and pancreatic ductal cancer [10,15]. In addition, entotic figures have been proposed to be an independent prognostic factor for resectable esophageal squamous cell carcinoma, as patients with a higher density of entotic structures have tended to have a longer postoperational survival time [7]. However, the prognostic value of entosis in tumors remains to be assessed.

In cell cultures, entosis is associated with loss of cell-to-cell and cell-to-matrix connections. In solid tumors, the loss of cell-to-cell connections is also observed during the initiation of metastasis, which is preceded by an epithelial–mesenchymal transition (EMT) [16]. During an EMT, carcinoma cells obtain certain characteristics of mesenchymal cells by losing E-cadherin expression and acquiring vimentin expression [17]. Thus, such cells lose their strong adherent phenotype and migrate to form secondary tumors. Moreover, cell-to-cell connections are disrupted during cell division; thereby, mitosis can also induce cell internalization [3]. This specific form of mitosis is referred to as entotic mitosis. Mitotic cell division of the inner entotic cell (as the prosurvival fate of entosis) has been frequently observed in cell cultures [2,10]. During the cell cycle, cells in the G2 phase are three times more active in forming cell-in-cell structures than G0/G1 resting cells are [18].

The mechanism of formation of entotic figures includes the induction of cell detachment by unidentified triggers, which is followed by strong cell-to-cell adhesion via E-cadherin and β-catenin. Subsequently, Rho/ROCK and actomyosin complexes are formed in one of the interacting cells, making it stiffer. This cell is pushed into the engulfing (outer) cell, characterized by the lack of activation of Rho-pathways [2,10]. It is difficult to extrapolate these in vitro observations to a similar scenario in human cancer tissues. Additionally, there are no published histopathological studies on entotic figures in clinical human cancer samples combined with an analysis of proliferation markers, such as Ki67.

Breast cancer is the most frequently diagnosed malignancy and the most common cause of cancer-associated death in women [19]. In diagnostic immunohistochemistry, breast cancer is characterized based on the expression of estrogen and progesterone hormone receptors (ER and PR, respectively) and the human epidermal growth factor receptor 2 (HER2) [6,9,20,21]. Another routine diagnostic marker of cell proliferation is Ki67 [22], which served as an indicator of proliferation in the presented study. Nevertheless, there is still a need to search for prognostic factors in breast cancer, e.g., PD-L1, which is suggested to be a valuable indicator in advanced triple-negative breast cancer [23]. However, the utility of PD-L1 as a predictive biomarker in other breast cancer subtypes remains unclear [24].

In the context of breast cancer, it has been reported that prognosis can differ depending on the tumor subtype [5]. Recently, in a cohort of 147 breast cancer patients, mainly patients with T1 tumors, entotic figures were found in 61.2% of the examined cases. In this cohort, entoses were associated with a favorable prognosis for local recurrence-free and disease-free survival. However, entosis-positive patients had an unfavorable prognosis in regard to metastasis-free survival, with the highest prognostic value being found in young patients [25].

Here, we analyzed and characterized entotic figures in a tissue microarray of 50 selected, entosis-positive breast cancer cases, with specimens being paired into primary lesions and corresponding lymph node metastases (100 tissue samples). We also performed an in-depth analysis of a case of double-negative, HER2-positive breast cancer (ER-, PR-, and HER+++) with a special focus on the occurrence of selected EMT hallmarks. Routine diagnostic immunochemistry (E-cadherin/vimentin and Ki67 immunostaining) was used for histopathological diagnostics.

## 2. Results

### 2.1. Characteristics of the Breast Cancer Cohort

The three following inclusion criteria were applied: (1) a histopathological diagnosis of NOS cancer, (2) lymph node metastases, and (3) the presence of entotic figures in the primary lesion and/or lymph node metastases. For the study, we selected a tissue microarray containing entotic figure-positive tissues from paired primary lesions and corresponding lymph node metastases from 50 women of a median age of 50 years old (47 NOS carcinoma cases and 3 ductal carcinomas). For the statistical analysis, only NOS carcinomas were included. Entotic figures were found in all the analyzed primary lesions and metastatic tissues. The detailed characteristics of the cohort are displayed in Table 1.

### 2.2. Analysis of the Breast Cancer Patient Cohort 

**Figure 1:**
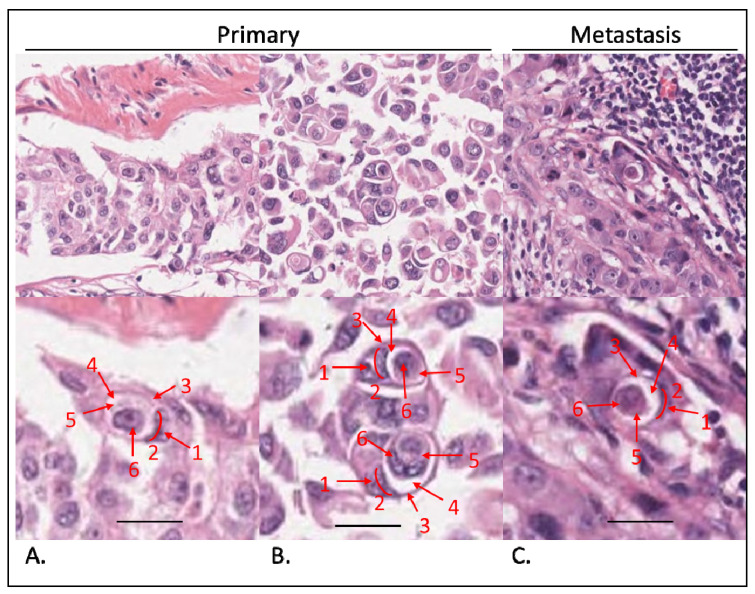
Evaluation of entotic structures in the cohort. Tissue samples stained with hematoxylin and eosin. (**A**,**B**) Entoses identified in primary tumors. Top panels observed under a 40× microscope objective; bottom panels digitally magnified to demonstrate Mackay’s criteria: (1) nucleus of the outer cell; (2) semilunar shape of the outer cell nucleus; (3) outer cell cytoplasm; (4) entotic vacuole; (5) inner cell cytoplasm; (6) inner cell nucleus. At least 4 out of 6 criteria have to be visible to classify a structure as an entotic figure. (**C**) Entosis in a lymph node metastasis. Top panel: 40× microscope objective; bottom panel is magnified and structures are marked as in (**A**,**B**). Scalebar: 15 µm.

Entotic structures were present in all the investigated cases (Figure 1). We investigated the correlation between the frequency of entotic figures and individual clinical parameters across the cohort, as well as that in the subgroup of patients sharing immunohistochemical characteristics with the selected case (which was ER-negative, PR-negative, and HER2-positive). The density of entotic figures was determined by assessing the surface area of the tumor tissue and rejecting areas of necrosis, fibrosis, or steatosis.

**Figure 2:**
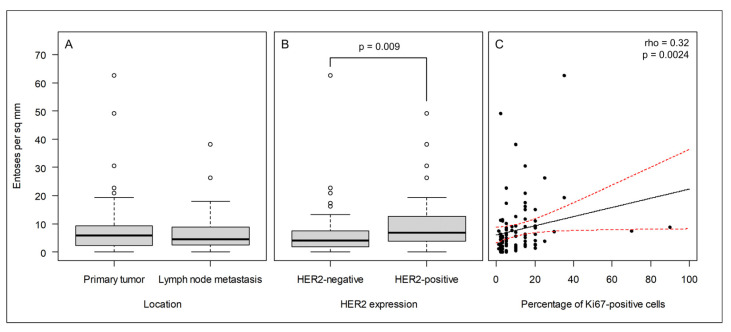
In the cohort of breast cancer patients, entosis correlates with the HER2 and Ki67 cancer markers. (**A**) The frequency of entoses does not differ between primary tumors and lymph node metastases; (**B**) the frequency of entoses is significantly higher in HER2-positive cancers than in HER2-negative cancers; (**C**) the frequency of entoses correlates with Ki67 expression. The black and red lines indicate the best linear fit and its 95% confidence interval, respectively.

As displayed in Table 2, across the cohort, we did not find significant differences between entotic figure frequency with regard to age, grading, staging, or expression of ER and PR receptors. In the case of PR expression, the *p*-value was established to be 0.037 in the metastases. However, we applied a more stringent *p*-value (*p* < 0.01) as the limit of statistical significance in this study. We did not observe a statistically significant difference in the entotic density between the paired primary lesions and corresponding metastatic tissues (Figure 2A). However, a statistically significant difference was observed for the HER2 receptor and Ki67 expression in correlation to the entotic figure number. Interestingly, the correlation with Ki67 occurred mostly in primary tumors, whereas the correlation with HER2 occurred mostly in lymph node metastases (Table 2).

In the case of the HER2 receptor and Ki67, increased expression was accompanied by an increased number of entotic figures in the investigated samples; therefore, the correlation was found to be positive in both cases (Figure 2B,C).

### 2.3. Entosis In Situ in a Double-Negative HER2-Positive NOS Breast Cancer Case

In order to better characterize entotic figures in tumors, after the macroscopic examination of postoperative tissues (described in Materials and Methods, Section 4.4), we performed a descriptive immunohistochemical analysis of the Ki67 proliferation marker and selected epithelial–mesenchymal transition markers (vimentin, E-cadherin) in the studied case of double-negative HER2-positive NOS breast cancer.

#### 2.3.1. Distribution of Entotic Figures in the Primary and Metastatic Tissue

**Figure 3:**
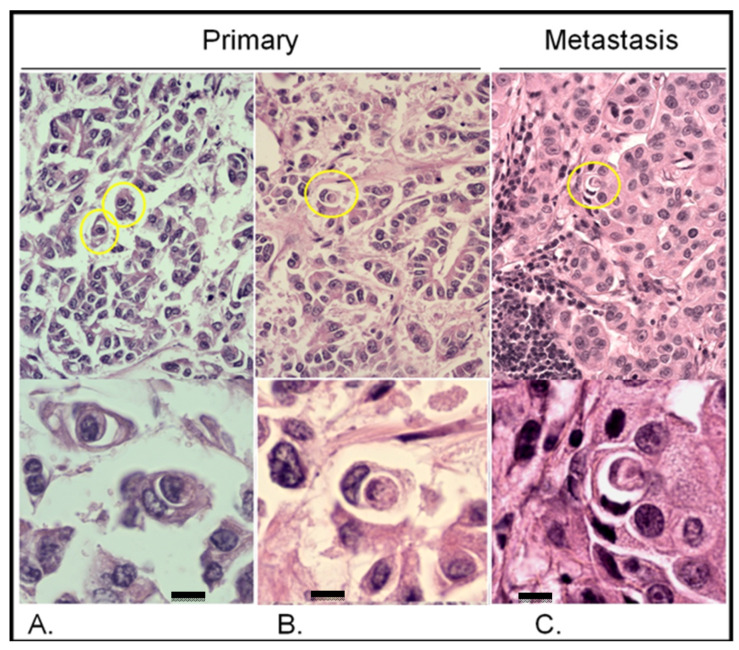
Entotic figures in the primary lesion and lymph node metastasis. The slides were stained with hematoxylin and eosin. (**A**,**B**) Entotic figures in the primary lesion. Top panels present entoses observed under a 10× microscope objective (marked using yellow circles) and the same cell-in-cell structures are displayed in a higher resolution (bottom panels; 40× microscope objective). (**C**) Lymph node metastasis: upper panel—10× microscope objective; lower panel—40× microscope objective. Scalebar 10 µM.

As shown in Figure 3, entotic cells (both cell-in-cell and multicell structures) were observed in all the examined slides in both the primary lesion and lymph node metastasis. The distribution of entotic figures was not homogeneous, and hotspots with an increased number of entoses were observed. These hotspots were located in low-differentiated parts of the primary lesion that did not present an organotypic structure (Figure 3A,B). Hence, there were no entotic figures in the more differentiated regions of the cancer characterized by a tubular structure. In the lymph node metastasis, entotic figures were observed mainly in subcapsular regions (Figure 3C).

#### 2.3.2. Analysis of the Correlation between E-Cadherin Yield and Entotic Structures

**Figure 4:**
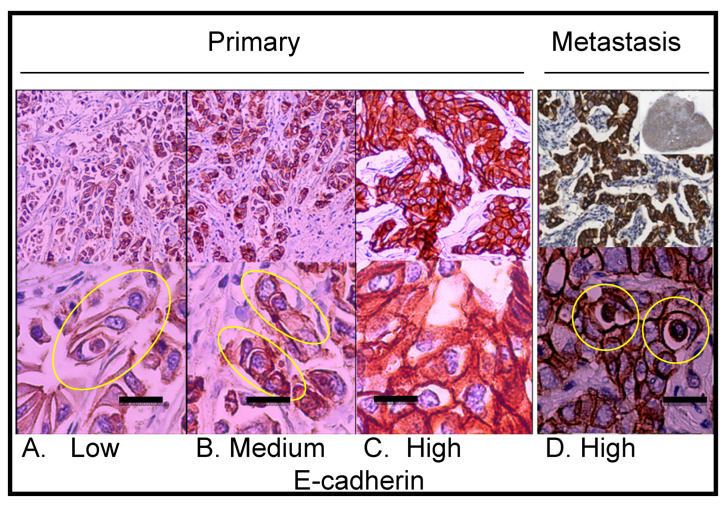
Immunohistochemical staining showing E-cadherin expression in the primary lesion and lymph node metastasis. (**A**) Primary lesion: region with low E-cadherin expression; the yellow ellipse indicates a multicell entotic figure. (**B**) Primary lesion: region with medium E-cadherin expression; the yellow ellipses indicate entotic figures. (**C**) Primary lesion: region with high E-cadherin expression; no visible entotic figures. (**D**) Lymph node metastasis; the upper right small insert: whole lymph node scan; strong and homogenous E-cadherin expression was observed within metastatic carcinoma cells; the yellow rings indicate entotic figures. Upper panel—10× microscope objective; lower panel—40× microscope objective. Scalebar 15 µM.

Immunostaining revealed that the epithelial marker E-cadherin was not homogenously expressed in the primary lesion. As shown in Figure 4, entotic cells were localized preferably in regions with a low-to-medium expression of E-cadherin (Figure 4A,B). Interestingly, no entotic figures were found in regions of high E-cadherin expression (Figure 4C). In contrast to the entotic cell occurrence pattern observed in the primary lesion, in the lymph node metastasis, a positive correlation was found between the E-cadherin yield and the number of entotic figures (Figure 4D).

#### 2.3.3. In the Analyzed Case, Entotic Figures Are More Frequent in the Lymph Node Metastasis 

In order to evaluate the number of entotic figures in the tissue specimens, we analyzed randomized fields acquired by whole specimen scanning using a histological scanner, and counted the number of entoses according to Mackay’s criteria. The total number of entotic figures calculated from the specimen scans was 2.15-fold higher in the lymph node metastasis than in the primary lesion (a mean of 5.8 vs. 2.7%; *p* < 0.0001) (Table 3).

#### 2.3.4. Descriptive Distribution of Entotic Figures in the Primary and Metastatic Breast Cancer Tissue

As shown in Figure 3 and Figure 4, entotic cells (both cell-in-cell and multicell structures) were observed in all the examined slides of both the primary lesion and lymph node metastasis. The distribution of entotic figures was not homogeneous and hotspots with an increased number of entoses were observed. These hotspots were localized in low-differentiated parts of the primary lesion that did not present an organotypic structure (Figure 3A,B). Hence, there were no entotic figures in the more differentiated regions of the cancer characterized by a tubular structure. In the lymph node metastases, entotic figures were observed mainly in subcapsular regions (Figure 3C and Figure 4D).

**Figure 5:**
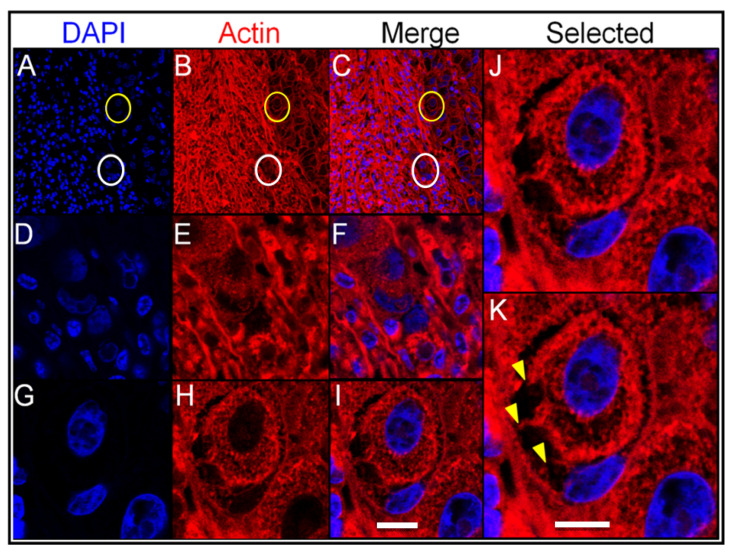
Confocal analysis of entotic figures in the lymph node metastasis. (**A**–**C**) A general view of the entotic cells stained with DAPI (nuclei; panel (**A**)) and phalloidin Atto-647N (actin; panel (**B**)); panel (**C**) shows panels (**A**,**B**) merged; circles indicate entotic figures; 10× microscope objective. (**D**–**F**) Magnified images of the entotic figure from panels (**A**–**C**) (white circle); entotic cells stained with DAPI (nuclei; panel (**D**)) and phalloidin Atto-647N (actin; panel (**E**)); panel (**F**) shows panels (**D**,**E**) merged; 40× microscope objective. (**G**–**I**) Magnified images of the entotic figure from panels (**A**–**C**) (yellow circle); entotic cells stained with DAPI (nuclei; panel (**G**)) and phalloidin Atto-647N (actin; panel (**H**)); panel (**I**) shows panels (**G**,**H**) merged; 40× microscope objective. (**J**,**K**) Magnified images of the entotic figure from panel (**I**). The arrowheads (panel (**K**)) indicate cytoplasmic bridges between the inner and outer entotic cell; 40× microscope objective. Scalebar 10 µM.

In order to study cell-to-cell interactions within entotic structures, a confocal microscopy analysis of the lymph node metastasis was performed. As shown in Figure 5 (panels A–C), entotic figures, which were identified by a characteristic nuclear shape (Figure 5D,G) and the presence of an entotic vacuole (Figure 5E,H), were located in the subcapsular region. A detailed analysis also revealed characteristic cytoplasmic bridges between the inner and outer entotic cell within the entotic vacuole (Figure 5J,K).

#### 2.3.5. Correlation between Vimentin Expression and Entotic Structure Frequency

**Figure 6:**
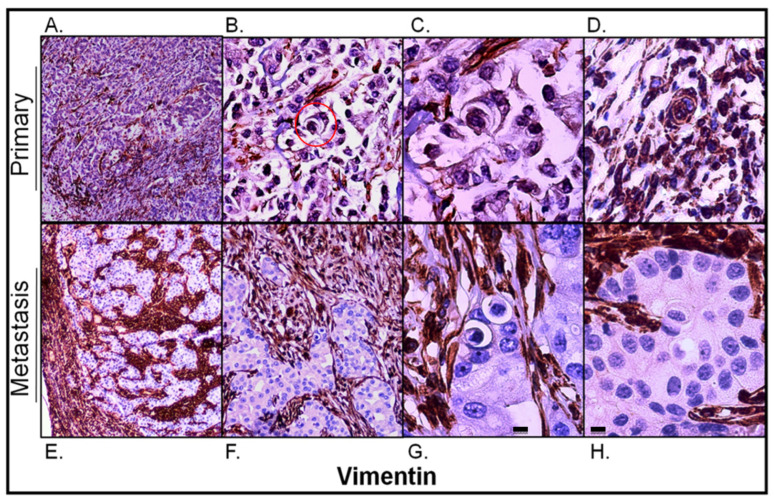
Vimentin expression in the primary lesion and lymph node metastasis. (**A**) Primary lesion: a general view; vimentin immunostaining is visible mainly in the stroma of the tumor; 4× microscope objective. (**B**) Primary lesion: a selected view with carcinoma cells slightly positive for vimentin; the red circle indicates a multicell entotic figure; 10× microscope objective. (**C**) Primary lesion: a 4-cell entotic figure in which all cells are immunoreactive for vimentin; 40× microscope objective. (**D**) Primary lesion: a part with higher vimentin immunoreactivity; a multicell entotic figure with at least two crescent-shaped nuclei is visible in the middle. (**E**) Lymph node metastasis: a general view, showing the subcapsular region; 4× microscope objective. (**F**) Lymph node metastasis: selected view with entotic figures; carcinoma metastatic cells are completely negative for vimentin; 10× microscope objective. (**G**,**H**) Lymph node metastasis: selected entotic figures; 40× microscope objective. Scalebar 10 µM.

Vimentin immunostaining was used as an EMT marker of epithelium-derived cancer cells to visualize connective tissue. As shown in Figure 6A, vimentin immunostaining was found mainly in the interstitial tissue of the primary lesion. However, vimentin-positive cancer cells were also observed (Figure 6B–D) and they corresponded to regions with a lower E-cadherin signal within them (compare to Figure 4A,B). Moreover, as shown in Figure 6C, entotic figures formed by vimentin-positive cells were of the same multicell morphology as those formed by cancer cells with medium E-cadherin expression (Figure 4B).

Unlike in the primary lesion, vimentin expression in the lymph node metastasis was strictly limited to the interstitial tissue corresponding to the connective tissue of the lymph node and its capsule (Figure 6E,F). The carcinomatous tissue of the metastasis was entirely negative for vimentin. All entotic figures found in the metastasis were also vimentin-negative (Figure 6G,H).

#### 2.3.6. Analysis of Proliferating Cells in Entosis

**Figure 7:**
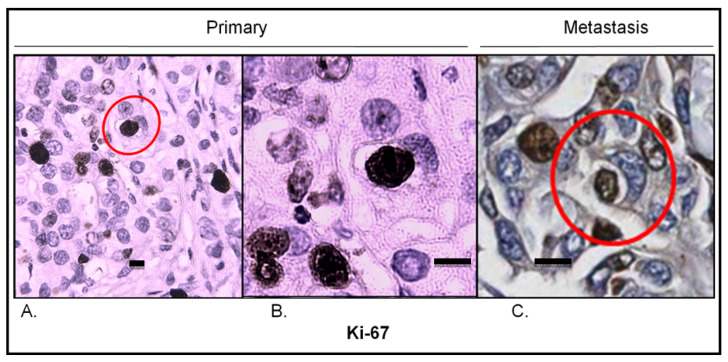
Ki67 expression in the primary lesion and lymph node metastasis. (**A**) Primary lesion: Ki67 hotspot; the red circle indicates an entotic figure with a Ki67-positive inner cell; 10× microscope objective. (**B**) A selected part of the Ki67 hotspot from panel A displaying a Ki67-positive inner cell in an entotic figure; 40× microscope objective. (**C**) Lymph node metastasis: Ki67 hotspot; the red circle indicates an entotic figure with a Ki67-positive inner cell; 20× histological scan. Scalebar 10 µM.

To visualize proliferating cells, Ki67 immunostaining was conducted. In the primary lesion, Ki67 hotspots corresponded to entotic hotspots (Figure 7A). Ki67-positive nuclei were identified in both entotic and nonentotic cells. Moreover, Ki67-positive internal entotic cells were present in both the primary lesion (Figure 7A,B) and metastasis (Figure 7C).

#### 2.3.7. Analysis of Vessel Markers and Entotic Figures

**Figure 8:**
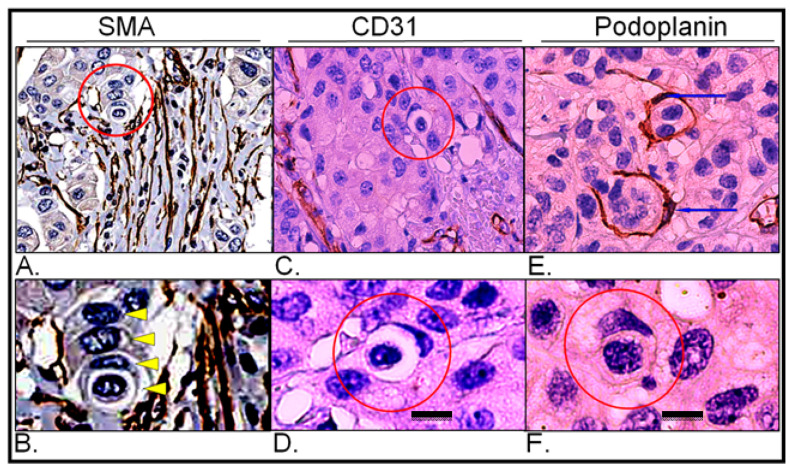
Expression of vascular markers in the primary lesion and lymph node metastasis; (**A**) Primary lesion: smooth muscle actin immunohistochemistry; the red circle indicates a multicell entotic figure; histological scan. (**B**) Primary lesion: smooth muscle actin immunohistochemistry; the yellow arrowheads display particular cells within a multicell entotic figure; digital magnification of panel A. (**C**) Primary lesion: CD31 immunohistochemistry; the red circle indicates entotic figures; 10× microscope objective. (**D**) Primary lesion: CD31 immunohistochemistry; view of selected entotic figure; 40× microscope objective. (**E**) Lymph node metastasis: podoplanin immunohistochemistry; the blue arrows indicate nuclei of lymph vessels; 10× microscope objective. (**F**) Lymph node metastasis: podoplanin immunohistochemistry; the red circle indicates entotic figure; 40× microscope objective. Scalebar 10 µM.

To exclude the possibility of misinterpreting entotic figures as cells within lymphatic or blood vessels, immunostaining for classical markers of vessels SMA, CD31 and podoplanin, was performed. As shown in Figure 8, entotic structures do not represent cells in either blood or lymphatic vessels and are rather found in low-vascular regions of both the primary lesion and lymph node metastasis.

## 3. Discussion

Entosis represents a type of cell-in-cell structure that can be found within a normal epithelium, but is much more prevalent in cancerous tissues [2,10]. Nowadays, entotic figures are considered an important and independent prognostic factor in many cancers; however, the type of prognosis can differ among various types of malignancies [10]. Recently, it was shown that in early hormone-sensitive breast cancer, entotic figures are an unfavorable prognostic marker in regard to metastatis-free survival [25]. While the mechanisms of entosis are well-characterized in cell culture studies in vitro [2,3,6,10], very little is known about the role and molecular factors that promote the formation of entotic structures in human cancers in vivo [10]. Therefore, we aimed to better characterize entoses in breast cancer using immunohistochemical methods.

E-cadherin staining helps to distinguish entotic figures from heterotypic cell-in-cell structures [8]. Therefore, in entosis, which is a type of homotypic cell engulfment, both cells should be E-cadherin positive, due to their epithelial origin (Figure 4). Additionally, entotic figures have to be distinguished from cells within vessels, which are surrounded by the endothelium. This can be achieved by immunohistochemical staining for blood vessel markers (CD31, SMA) [26,27] and lymphatic endothelial markers (podoplanin) [28] (Figure 8).

In the described cohort, we only analyzed NOS breast cancers positive for entotic figures. The obtained data indicate that entosis is generally independent from patient age, grading, staging, tumor size and hormone receptor status.

However, two frequently used markers of breast cancer, Ki67 and HER2, turned out to be significantly correlated with the presence of entotic structures (Table 3; Figure 3B,C). This finding from the cohort study was confirmed in the selected case, which was studied in more detail, resulting in the observation of entotic hotspots overlapped with Ki67-positive hotspots (Figure 7).

Ki67 is a well-known diagnostic marker of cell proliferation in cancers and is frequently used for the immunohistochemical characterization of breast cancer [22]. The observation that entotic hotspots correspond with Ki67 hotspots is in accordance with the recently published data, in which it was presented that cells in the G2 phase of the cell cycle are more likely to form entotic figures than cells in the resting, G1/G0 phase are [18]. Furthermore, during each cellular division, cells detach from neighboring cells, and therefore, mitosis can favor cell internalization through entosis [3]. For the first time in the reported literature, we have identified a Ki67-positive inner entotic cell in situ in a tumor (Figure 7). Until now, mitosis of engulfed cells has only been reported in cell culture models [2]. Thus, our findings suggest that “entotic mitosis” can take place not only in cell cultures, but also in human cancers in vivo. Although we were not able to visualize mitotic figures of the internal entotic cells, their Ki67-positivity suggests that the internal entotic cell can be engaged in cell cycle progression. Moreover, correlation between entosis and Ki67 might indicate that entosis is a mechanism protecting inner cells from environmental factors, including immune response or molecular-based targeted therapeutics. Ki67-positive inner entotic cells are potentially able to divide. We hypothesize that such cells can produce a more malignant phenotype that may be responsible for the relapse and/or recurrence of cancer.

HER2 is a classical diagnostic marker in breast cancer [29]. HER2 overexpression is consistently associated with a high tumor grade, aneuploidy, a high cell proliferation rate, and alterations in a variety of other molecular biomarkers associated with a more malignant phenotype [30]. Therefore, the correlation between the presence of entotic structures and HER2 overexpression suggests that entotic figures can also be associated with a more malignant cell phenotype.

The correlation of entosis with both Ki67 and HER2 expression connects entosis with cell proliferation. During mitosis, cells detach from neighboring cells, and therefore, mitosis can facilitate cell internalization through entosis [3]. It has been shown that epithelial cells need to be detached from the extracellular matrix to initiate the formation of entotic figures in vitro [2]. In vivo, such conditions can be found in low-differentiated tumor areas. In contrast, in more differentiated, organotypic (tubular) structures, cancer cells strongly adhere to neighboring cells, which may limit their ability to form entoses. This is consistent with our findings showing that entotic hotspots only occur in low-differentiated parts of the primary lesion. Furthermore, this is also in accordance with the observation that a higher entotic ratio can be observed in regions characterized by low-to-medium E-cadherin expression. The level of E-cadherin expression is related to the grade of differentiation of invasive ductal breast cancer and its loss is a hallmark of EMT [31]. E-cadherin is responsible for the strong cell-to-cell adhesion that is observed in a well-differentiated epithelium. Thus, organotypic cancer structures, which are strongly positive for E-cadherin, are expected to limit the formation of entoses. In this context, here we found that entosis correlates with Ki67 expression in NOS breast cancer, while other authors demonstrated that Ki67 correlates with a loss of E-cadherin expression in head and neck squamous cell carcinoma [32]. Moreover, in head and neck cancers, entosis is a predictive marker associated with worse prognosis [12].

During metastasis, cancer cells undergo an EMT, which increases the expression of vimentin, and the expression of E-cadherin is switched to that of N-cadherin [8]. The expression of N-cadherin is a classical sign of an EMT; however, N-cadherin is not routinely used in breast cancer diagnostics [33]. Thus, we considered increased vimentin and reduced E-cadherin expression indicators of an EMT. After the EMT, cells are able to migrate from the primary lesion into lymphatic vessels, reaching regional lymph nodes [34]. In lymph nodes, they undergo the reverse process to the EMT, a mesenchymal to epithelial transition (MET), in which cells reacquire E-cadherin and lose vimentin expression, and thereby strong adherence is reconstituted [35]. In the lymph node metastasis, we found that entotic figures were visible within tissue sections presenting a high expression of E-cadherin and low vimentin expression. This suggests that entoses in metastases may be initiated in semiadherent conditions, before or during MET. Additionally, metastatic cells (after the process of MET) formed numerous entotic figures, the number of which was significantly higher than in the primary lesion.

Our findings indicate that in the investigated cohort, entotic structures do not occur more frequently in metastases (Table 3). In contrast, in the investigated whole lymph node specimen of the double-negative, HER2-positive, NOS breast cancer case, the number of entotic figures was statistically higher than that in the primary lesion. It should be noted that the majority of entoses were localized in the subcapsular region of the lymph node, and the tissue microarrays used for the cohort study often lacked this part of the node.

Limitations of the study include the fact that our study only included cases of NOS breast cancer (TNM II and III) positive for entoses. Moreover, our study is a histopathological analysis; thus, we are unable to correlate the number of entotic figures with the survival of patients due to a lack of clinical data. Nevertheless, for the first time, we showed that entosis does not depend on the hormonal status of the cancer, but correlates with two prognostic factors: Ki67 and HER2.

## 4. Materials and Methods

### 4.1. Preparation of the Human Breast Cancer Cohort

For a better understanding of the pattern of entoses in breast cancer, an analysis of human tumor tissue microarray (TMA) slides (core diameter: 1.5 mm; TissueArray.Com LLC, formerly US Biomax; Derwood, MD, USA) was performed.

The sample size was estimated with the G*Power software (version 3.1.9.7, Heinrich-Heine-Universität Düsseldorf, Düsseldorf, Germany), as described previously [36]. The number of specimens was 106 (calculation for effect size = 0.5, α = 0.05, and β = 0.2). In total, 100 tissue specimens from a cohort of 50 breast cancer patients were analyzed. For every patient, tissue samples from both the primary tumor and lymph node metastases were assessed. Additionally, one pheochromocytoma core was included as a control.

For each analyzed case, information regarding age, grading, staging, TNM status, tumor diameter, as well as the expression of Ki67, HER2, ER, and PR receptors was obtained. Detailed data regarding each sample plotted on the slide are provided on the company’s website (https://www.tissuearray.com/tissue-arrays/Regional_Lymph_Nodes_Metastasis/BR10010f, assessed on 1 October 2022).

All tissues were collected under the highest ethical standards with informed consent having been obtained from each patient (National Human Genetic Resources Sharing Service Platform: 2005DKA21300). The entotic figures within the cohorts were analyzed by four independent researchers, including a certified clinical pathologist, who also verified the specimens. For a coherent evaluation of entoses, the following criteria established by Mackay et al. [8,9] were defined: complete encirclement of the inner cell by the host cell membrane, a round shape of the inner cell, and a semilunar nucleus displaced to the margin of the host cell.

### 4.2. Preparation of the Cohort of Histopathological Specimens

In order to ensure the proper quality of the clinical material, standard histopathological procedures were applied. The surgical breast cancer samples (TissueArray.Com/US Biomax) were fixed within 30 min after surgery in 10% neutral formalin, then dehydrated in ethanol, cleared with xylene and embedded in paraffin using a tissue processor (Leica; Wetzlar, Germany). Afterwards, each tissue specimen was individually examined by a certified pathologist and assessed according to the standardizations of diagnosis, classification and pathological grade published by the World Health Organization (WHO). Standard immunohistochemistry (IHC) protocols were performed to ensure the accuracy and specificity of the tissue array products. After deparaffinization and washing in phosphate-buffered saline (PBS), the antigen retrieval step was performed by heating the slides in a 0.01 M sodium citrate buffer (pH 6.0) for 15 min. Next, the specimens were washed in PBS and blocked using normal goat serum for 20 min at RT. Then, an appropriate primary antibody (Table 4) was applied for 1 h at RT, followed by rinsing the slides in PBS and incubating them automatically with a cocktail of HRP-labeled secondary antibodies together with diaminobenzidine from Ventana UltraView Universal DAB Detection Kit (Roche Diagnostics; Risch-Rotkreuz, Switzerland) or manually by the standard ABC technique using a biotin-conjugated secondary antibody and 3,3′-Diaminobenzidine (DAB) Liquid Substrate System tetrahydrochloride (Sigma-Aldrich; Darmstadt, Germany). The degree of staining was controlled using a light microscope. After the final wash in PBS, the slides were stained with hematoxylin. After the array was dehydrated and achieved transparency, it was mounted using the Vectastain ABC system (Vector Laboratories, Inc.; Newark, CA, USA).

Each collected specimen was consented to be used by both the hospital and the individual. An informed consent form was obtained from every patient, and the rights to hold the research for any purpose or for further commercialized uses were waived.

### 4.3. Evaluation of Entoses in the Cohort

The histopathological specimens were digitalized and analyzed with the use of a self-developed online platform. Each specimen was examined by three independent researchers, who marked entotic structures, according to Mackay’s criteria, on the images. Each selected entotic figure was verified by a separate team consisting of three researchers. For statistical analyses, all necrotic and noncancerous tissue areas were excluded from further studies, and the surface area of cancerous tissue was determined using the self-designed platform. The frequency of entoses was calculated as the number of entotic structures per square millimeter of cancerous tissue.

The correlations (patient age and percentage of Ki67-positive cells) were determined using Spearman’s rank correlation test. The differences between groups (e.g., in ER-, PR-, HER2-positivity, TNM, and grade) were assessed using the Mann–Whitney U test (for two groups) or the Kruskal–Wallis test (for more than two groups). The paired Wilcoxon test was used to test for differences in the frequency of entoses between primary tumors and their corresponding lymph node metastases. Statistical significance was considered at a *p*-value of <0.05. All calculations were performed using the R Project for Statistical Computing software (R Core Team (2020). R: a language and environment for statistical computing. R Foundation for Statistical Computing, Vienna, Austria; (https://www.R-project.org/, accessed on 1 October 2022).

### 4.4. Preparation of Histopathological Samples from the Breast Cancer Patient

Archival paraffin tissue specimens from a 75-year-old Caucasian woman with double-negative HER2-positive breast cancer (pT4N3) were obtained.

The macroscopic image was as follows:

The right mammary gland with the contents of the axilla (1768.0 g; 24.0 × 22.0 × 5.5 cm) was obtained for examination. It was covered with an incised skin ellipse (21.0 × 18.0 cm) and the contents of the armpit (12.5 × 6.0 × 3.0 cm). At the border of the upper quadrants, an ulcerated, bulging tumor (10.5 × 7.5 × 11 cm) was found, with a thoracic margin of 1.5 cm, an axillary margin of 9.0 cm, and a medial margin of 6.0 cm. On the cross-section in the axillary cavity, a pack of fat-infiltrated axillary lymph nodes, measuring 7.5 × 5.5 × 3.5 cm, was identified. On the surface of the skin, an ulcer of a diameter of 1.0 cm was present. The preparations were composed of (1) the skin bump; (2) the tumor with a skin margin; (3) the central part of the tumor; (4) other parts of the tumor; (5) the lower quadrants of the mammary gland outside the tumor; (6) the upper quadrants of the mammary gland outside the tumor; (7) a bundle of lymph nodes; (8) a nipple from the side of the tumor.

The histopathological diagnosis was based on the following: invasive cancer NOS, NST NHG 3 (3 + 3 + 3)/28 mitoses/10HPF ER-, PR-, HER2+++, Ki67 positivity in 30% of nuclei, lymphangiosis carcinomatosa, and also present carcinoma ductale in situ foci with high nuclear atypia, constituting about 10% of the cross-sectional area of the tumor.

Both the primary breast lesion from the central part and the metastasis into the axillary lymph node, which was a single metastasis at the time of tissue sample collection, were examined.

The material underwent standard diagnostic histopathologic procedures, with HE staining and immunohistochemistry using DAKO EnVision™ + System (DAKO; Carpinteria, CA, USA). For the purpose of this study, the slides were specially immunostained for E-cadherin (an epithelial marker), SMA and CD31 (vascular markers), podoplanin (a marker of lymphatic vessels), and Ki67 (a marker of cell proliferation. All antibodies were from Roche Diagnostics; Switzerland; see Table 4). The slides were either scanned using an Aperio GT 450 histological scanner (Leica; Wetzlar, Germany) or analyzed using an optic microscope, Eclipse 50i (Nikon; Tokyo, Japan), equipped with a digital camera, MI6 (OPTA-TECH; Warsaw, Poland). The histological scans containing >10,000 cells were assessed by three independent researchers, including a certified clinical pathologist. The number of entotic figures was counted according to the diagnostic criteria established by Mackay [9].

The slides were also stained with DAPI for nuclei and phalloidin Atto-647N (Sigma-Aldrich; Darmstadt, Germany) for actin visualization, and analyzed by SP5 confocal microscopy (Leica; Wetzlar, Germany). The deparaffinized sections were incubated with Phalloidin-Atto (1:1000; Sigma-Aldrich; Wetzlar, Germany) for 1 h at RT, and subsequently the slides were embedded in VECTASHIELD Antifade Mounting Medium with DAPI (Vector Laboratories; Newark, CA, USA).

Data analysis was performed using the GraphPad Prism 6.0 (GraphPad; San Diego, CA, USA) software. For statistical purposes, a nonparametric Mann–Whitney U test was used. Statistical significance was considered at a *p*-value of <0.05.

## 5. Conclusions

Our results confirmed that homotypic entosis can be found in histopathological examinations of breast cancer, both in the primary lesions and lymph node metastases. In the cohort of NOS breast cancers, we found a positive correlation between the presence of entotic figures, and two well-known and routinely used predictive factors in breast cancer: Ki67 and HER2. For the first time, we identified Ki67-positive inner engulfed entotic cells in a tumor in situ. The presence of Ki67-positive internalized cells, as well as the positive correlation of entoses with the Ki67 proliferation marker, supports the formerly published data that indicate that the inner entotic cell can also divide. However, the fate of entotic cells may differ depending on not yet fully understood conditions. We observed certain immunohistochemical changes characteristic of an EMT (accompanied by entotic figures), which takes place during metastasis. Thus, our findings indicate that entotic figures in breast cancer can have potential diagnostic value, since they correlate with two classical prognostic factors of breast cancer.

## Figures and Tables

**Table 1:** Characteristics of the cohort of breast cancer patients.

Characteristic	Category	Number of Cases	Number of Cases % of Total
Age	Median	50	
	Range	28–80 42	84%
	20–60 Over 60	8	16%
Type	NOS Invasive Ductal Carcinoma	47	94%
	Invasive Lobular Carcinoma	3	6%
Grade *	1	10	20%
	2	28	56%
	3	8	16%
TNM	I	0	0%
	II	21	42%
	III	29	58%
Size (primary lesion)	<2 cm	1	2%
	2–5 cm	33	66%
	>5 cm	16	32%
Lymph node involvement	N1 N2	30 20	60% 40%
Immunohistochemical properties	ER **	18 pos/32 neg	36%/64%
	PR **	8 pos/40 neg	16%/80%
	HER2 ***	20 pos/30 neg	40%/60%
	Ki67 ****	8 high/38 low	16%/76%
Entotic structures	at least 1/sq mm	47/sq mm	100% of NOS
	of cancer tissue		

* Grade was not evaluated in 4 cases. ** ER and PR were defined as positive for ++ and +++ immunoreactivity. PR expression was not evaluated in 2 cases. *** HER2 was defined as positive for 2+ and 3+ immunoreactivity. **** Ki67 expression was defined as high for ≥20% of immunoreactive cells. Ki67 expression was not evaluated in 4 cases.

**Table 2:** Statistical analysis between the number of entoses and selected parameters in the cohort of breast cancer patients (*p*-values) (ρ—Spearman’s correlation coefficients).

Statistics of Entosis	All NOS		
Characteristic	Samples	Primary	Metastatic
Age (correlation) *	0.8402	0.6403	0.7603
	ρ = −0.02	ρ = −0.06	ρ = 0.04
Age (groups < 50 and >50 years old) **	0.8065	0.5733	0.6692
Primary vs. metastasis **	0.4927		
Primary vs. metastasis (paired test) ***	0.2839		
TNM ****	0.6743	0.8303	0.7729
T (from TNM) ****	0.9845	0.8232	0.8397
N (from TNM) ****	0.0517	0.1657	0.1812
Grade	0.2902	0.9562	0.2631
ER (2 grades) **^,#^	0.2058	0.8005	0.1569
PR (2 grades) **^,#^	0.0645	0.5428	0.0372
HER2 (2 grades) **^,#^	**0.0091**	0.2467	**0.0133**
Ki67 (correlation) *	**0.0024**	**0.0051**	0.0635
	**ρ = 0.32**	**ρ = 0.41**	ρ = 0.28

Association of patient and tumor characteristics with the number of entoses among 50 patients with metastatic NOS breast cancer. Numbers in the table represent the *p*-value, and numbers in bold indicate significant results (*p* < 0.01). * Spearman’s rank correlation test; ** U Mann–Whitney test; *** Paired Wilcoxon test; **** Kruskal–Wallis test; ^#^ differences assessed between positive and negative tumors, defined as in Table 1.

**Table 3:** Analysis of entotic figures calculated from slide scans (cells were positive for E-cadherin).

Entotic Figures	Primary Tumor	Metastasis
Mean (%)	2.68%	5.79%
Std. Error	0.2792	0.6178
*p* value	<0.0001	
Median	2.36%	5.16%
Minimum	0.78	0.49
Maximum	5.83	20.83
Total entosis counted	188	531
Total cells counted	7418	10,069
Number of fields	19	40

Statistical analysis was assessed using the Mann–Whitney U test (*p*-value < 0.05).

**Table 4:** Diagnostic primary antibodies used in the study.

Antigen	Type (Clone)	Colocalization	Source and Procedure
Ki67	Rabbit Monoclonal (30-9)	Nuclear	For all, Roche Diagnostics
E-cadherin	Mouse Monoclonal (36)	Membranous	Standard histochemical routine using the BenchMark ULTRA system (Roche) and standard buffers and solutions
Vimentin	Mouse Monoclonal (V9)	Cytoplasmic	
HER2	Mouse Monoclonal (4B5)	Membranous	
ER	Rabbit Monoclonal (SP1)	Nuclear	
PR	Rabbit Monoclonal (1E2)	Nuclear	
CD31	Mouse Monoclonal (JC70)	Membranous/Cytoplasmic	
Actin, Smooth Muscle (SMA)	Mouse Monoclonal (1A4)	Cytoplasmic	
Podoplanin	Mouse Monoclonal (D2-40)	Membranous	

## Data Availability

Data available on request due to restrictions, e.g., privacy or ethical. The data presented in this study are available on request from the corresponding author.
